# Mid-term results of a new-generation calcar-guided short stem in THA: clinical and radiological 5-year follow-up of 216 cases

**DOI:** 10.1186/s10195-019-0537-z

**Published:** 2019-10-31

**Authors:** Karl Philipp Kutzner, Stefanie Donner, Lennard Loweg, Philipp Rehbein, Jens Dargel, Philipp Drees, Joachim Pfeil

**Affiliations:** 1 Department of Orthopaedic Surgery and Traumatology, St. Josefs Hospital Wiesbaden, Beethovenstr. 20, 65189 Wiesbaden, Germany; 2 Department for Orthopaedics and Traumatology, University Medical Centre of the Johannes Gutenberg University Mainz, Mainz, Germany

**Keywords:** Short-stem, Total hip arthroplasty, Optimys, Mid-term, Subsidence, Bone remodelling, Stress-shielding

## Abstract

**Background:**

In recent years, a variety of short stems have been introduced. To date, mid- and long-term results of calcar-guided short-stem designs have been rarely available.

**Materials and methods:**

Two hundred and sixteen calcar-guided short stems were included in combination with a cementless cup in a prospective study. Patients were allowed full weight-bearing on the first day postoperatively. Harris hip score (HHS) as well as pain and satisfaction on visual analogue scale (VAS) were assessed during a median follow-up of 61.7 months. Standardised radiographs were analysed at predefined time points regarding radiological alterations such as bone resorption and remodelling, radiolucency, osteolysis and cortical hypertrophy using modified Gruen zones.

**Results:**

At mid-term follow-up, no revision surgery of the stem had to be performed in the whole collective. At 5 years, HHS was 97.8 (SD 4.7), satisfaction on VAS was 9.7 (SD 0.7), rest pain on VAS was 0.1 (SD 0.5), and load pain on VAS was 0.6 (SD 1.2). Compared to the 2-year results, femoral bone resorption increased significantly at the 5-year follow-up (3.9% versus 42.3%). Rate of femoral cortical hypertrophy remained stable, occurring in a total of 9 hips (4.5%). At the 5-year follow-up, 2 stems (1.0%) showed non-progressive radiolucent lines with a maximum width of 2 mm. Signs of osteolysis were not observed. Compared to the 2-year follow-up, no further subsidence was observed.

**Conclusions:**

The rate of stem revision (0%) at the mid-term follow-up was remarkable and indicates the principle of using a calcar-guided short stem as being a safe procedure. However, signs of bone-remodelling, indicating some amount of stress-shielding, must be acknowledged at 5 years depending on stem alignment and type of anchorage.

**Level of evidence:**

IV, Prospective observational study

*Trial registration* German Clinical Trials Register, DRKS00012634, 07/07/2017 (retrospectively registered)

## Introduction

To date, short-stem total hip arthroplasty (THA) is an increasingly established procedure. Short stems present as a bone and soft-tissue preserving alternative to conventional stems and offer the opportunity for revision with a standard length stem if needed [1]. Studies have shown a less pronounced loss in bone mineral density around the proximal femur [2–4] and similar risks of revision compared to conventional implants [5–7]. Migration patterns of short hip stems have been investigated with “Einzel-Bild-Roentgen-Analysis” (EBRA) and with radiostereometric analysis (RSA), confirming sufficient fixation and stable short- and mid-term osteointegration [8–11].

A great variety of short stems have been introduced to the market in the last decade, providing distinct differences in the design regarding stem length, level of osteotomy and insertion technique. Khanuja et al. proposed four categories of short stems: femoral neck only, calcar loading, lateral flare calcar loading and shortened tapered stems [12]. However, short stems of the newest generation cannot be easily classified, since they can be both calcar loading and diaphyseal anchoring, depending on the individual stem alignment according to the patient’s anatomy [13, 14]. Thus, particularly in Europe, the term “calcar-guided” short stems has been established [15]. Yet, the impact of design differences on bone remodelling is not fully understood [16].

In order to prove comparable lifetimes of new-generation short stems and conventional stems, clinical and radiological follow-up studies need to be carried out, which are not disassociated from first-time use and the learning curve that comes with a new implant and implantation concept [17].

Early clinical results of the newest generation of neck preserving, calcar-guided short stems are encouraging [18–20]. Recent studies also show a good capability of reconstructing the physiological hip anatomy in terms of maintaining the femoro-acetabular offset and a high variety of caput-collum-diaphyseal angles (CCD) [21–23]. At short-term follow-up, a low incidence of bony alterations indicates a stable and durable osteointegration and physiological load distribution [20, 24]. Short-term registry data from the Australian Orthopaedic Association National Joint Replacement Registry (AOANJRR) recently confirmed encouraging cumulative rates of revision in early stages [25].

At present, there is no data available on the long-term outcomes of calcar-guided short stems and mid-term results of different type 2 short stems, according to Khanuja et al., have rarely been published [26, 27].

The aim of this study was to assess clinical and radiological mid-term results of a new-generation calcar-guided short stem (optimys, Mathys Ltd., Bettlach, Switzerland) in a 5-year follow-up. This report updates the previously published short-term results as part of a prospective observational cohort study [20].

## Materials and methods

**Fig. 1:**
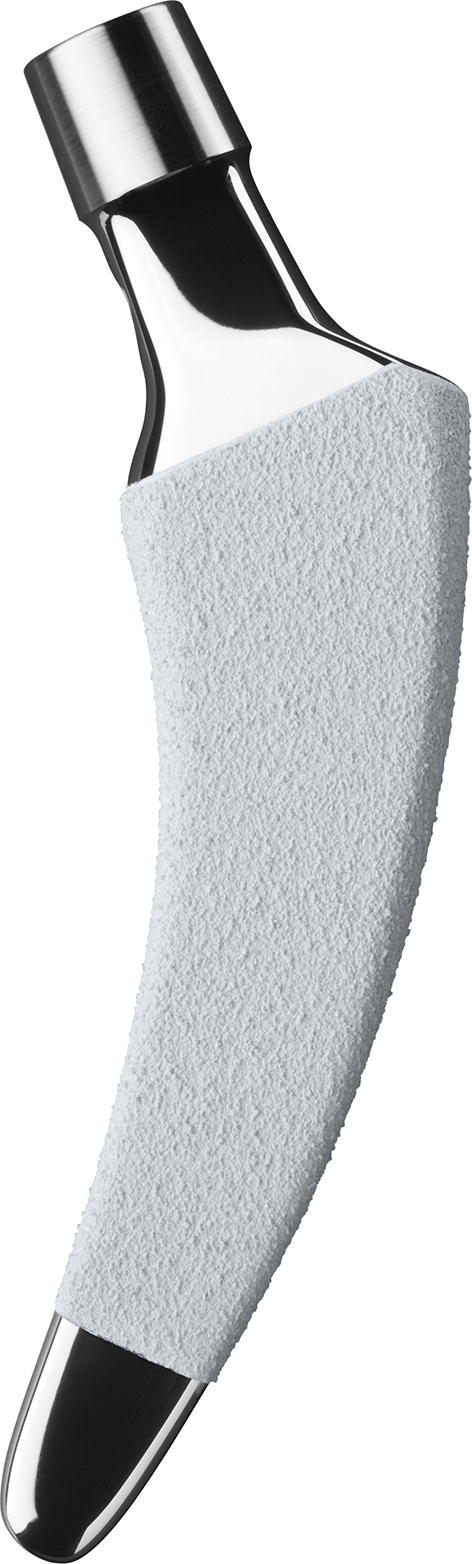
The optimys short stem (Mathys Ltd. Bettlach, Switzerland)

In the present prospective study, 216 consecutive cases in 162 patients were included. Surgery was performed in 73 women and 89 men. Median patient age was 63.5 years (range 33.4–88.0 years). In 54 patients, the treatment was bilateral simultaneously, 108 patients were operated unilaterally. In all patients the calcar-guided short stem optimys (Mathys Ltd. Bettlach, Switzerland) was used (Fig. 1). It has been rated type 2B in the classification system of Khanuja et al. [12]. In the Jerosch classification it accounts for the group of partially neck preserving short stems [28]. The optimys stem was combined with cementless press-fit cups (*n* = 177 Fitmore, Zimmer; *n* = 39 RM Pressfit vitamys, Mathys Ltd. Bettlach) with a ceramic-polyethylene bearing couple. The implantations were performed at a single centre, in the years 2010 to 2012. All operations were performed through a minimally invasive, antero-lateral approach in a standardised surgical technique [29]. The indications for implantation were as follows: 91.7% (*n* = 198) primary osteoarthrosis, 5.1% (*n* = 11) femoral head necrosis, 2.3% (*n* = 5) congenital dysplasia and 0.9% (*n* = 2) secondary osteoarthrosis. All patients started physiotherapy and were allowed full weight-bearing ambulation on the first day postoperatively.

The follow-up included a maximum of 7 time points: preoperative, during hospital stay, 6 weeks, 6 months, 1 year, 2 years and 5 years postoperatively.

Complications and potential reasons for revision surgery were documented during follow-up.

For clinical examination, the Harris hip score (HHS) as well as rest pain and load pain on visual analogue scales (VAS) were assessed at every follow-up time point.

For radiographic analysis all patients underwent standardised pre- and postoperative digital antero-posterior imaging according to Kutzner et al. [20]. Using a modification of the zones described by Gruen [30], bone resorption and remodelling, radiolucency, osteolysis and cortical hypertrophy were analysed in the radiograph after 5 years and were related to the 2-year results. To detect reduction of bone mineral density (BMD), the proximal femoral bone was scanned in order to find areas with enhanced bone transparency and thinned or resorbed trabeculae according to the Singh index [31]. Grades 1–3 were defined as bone resorption. Lucent lines were detected and the maximal width was measured. Cortical bone width was measured preoperatively and during follow-up in order to detect increase or decrease of width. All measures were obtained with the digital radiograph templating software MediCAD (Version 5; Hectec; Landshut, Germany). Magnification error was addressed using a ball with a known diameter as a scaling factor or the known diameter of the prosthetic femoral head as an internal reference.

The subsidence was digitally measured in a standardised technique using a coordinate system according to Kutzner et al. [20]. The measurements were done on the 5-year follow-up image and the subsidence was calculated taking into account the 2-year results. According to Bieger et al., measurements of at least 2 mm were considered reliable subsidence [32].

### Statistical analysis

Data were described by median and range or by mean and standard deviation (SD). For the comparison of the radiological results between 2 and 5 years, the McNemer’s test was used (2-sided). A *p* value < 0.05 was considered as indication for difference. All statistical analyses were performed using SAS Enterprise Guide 7.13 (SAS Institute Inc., Cary, USA).

## Results

**Fig. 2:**
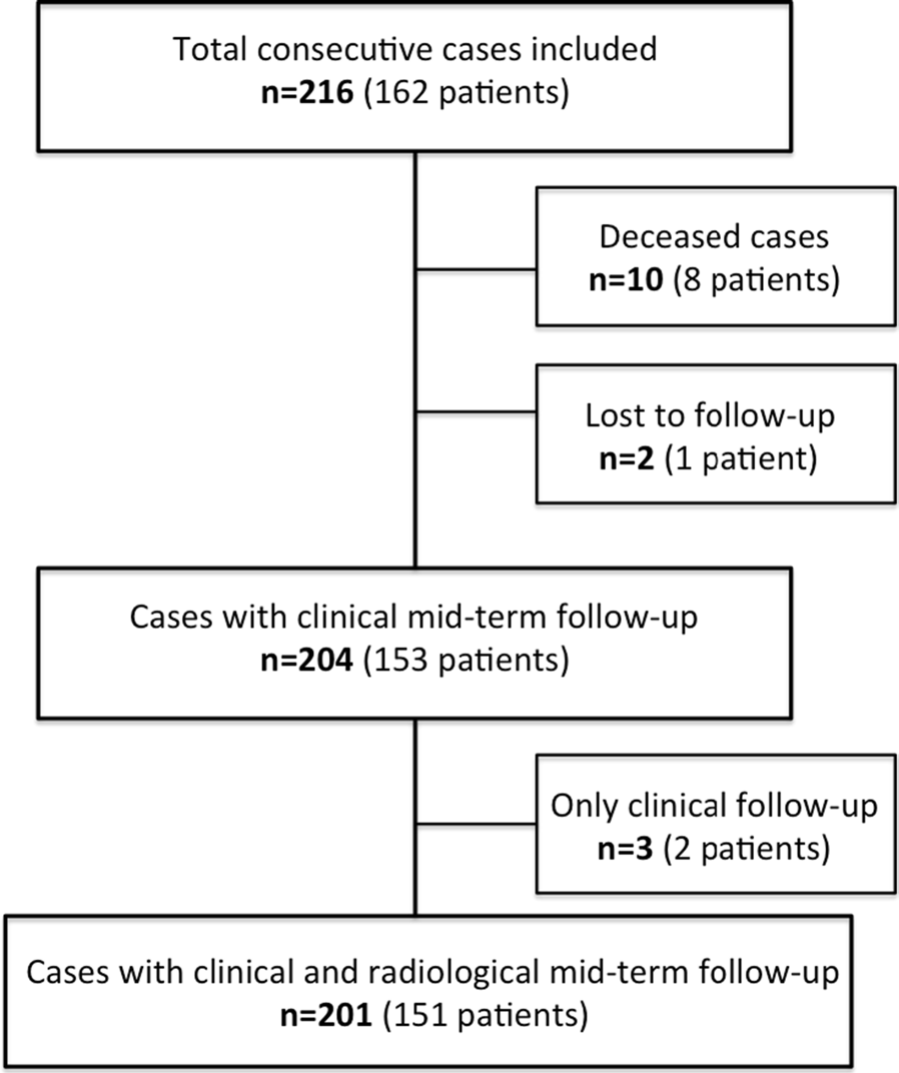
Flow-diagram of follow-up

After 5 years, 8 patients with 10 hips were known to be deceased with the investigated implants in situ. One bilateral patient was lost to follow up at mid-term. Thus, of the initially included 216 cases, 204 hips in 153 patients could be analysed after 5 years. In two patients with three hips, only a clinical follow-up could be performed (Fig. 2). The median follow-up time was 61.7 months (range 57.2–83.7 months). One early revision was performed due to early deep infection with change of head and inlay unrelated to the investigated implant. One intraoperative crack of the greater trochanter occurred, without any clinical malfunction. No therapy was required. One case of deep vein thrombosis (DVT) could be treated successfully. One traumatic dislocation occurred 3 years after surgery followed by closed reposition without any further therapy needed.

At the 5-year follow-up, no stem-related revision had to be performed in the whole cohort, resulting in a 100% survival rate.

Clinical results such as HHS as well as pain and satisfaction on VAS during follow-up are shown in Table 1.

**Table 1:** Clinical outcome over time

FU	*N*	Mean	SD	Min	Median	Max
VAS rest pain						
PreOP	216	5.2	3.0	0.0	5.0	10.0
6–12 weeks	211	0.6	1.2	0.0	0.0	6.0
6 months	186	0.2	0.8	0.0	0.0	5.0
12 months	191	0.3	1.0	0.0	0.0	7.0
24 months	202	0.2	0.7	0.0	0.0	7.0
5 years	204	0.1	0.5	0.0	0.0	3.0
VAS load pain						
PreOP	216	7.7	2.2	0.0	8.0	10.0
6–12 weeks	211	1.2	1.8	0.0	0.0	8.0
6 months	186	0.9	2.0	0.0	0.0	9.0
12 months	191	0.7	1.5	0.0	0.0	8.0
24 months	202	0.4	1.1	0.0	0.0	7.0
5 years	204	0.6	1.2	0.0	0.0	5.0
VAS satisfaction						
PreOP	216	1.8	2.2	0.0	1.0	10.0
6–12 weeks	211	9.4	1.1	0.0	10.0	10.0
6 months	186	9.4	1.3	1.0	10.0	10.0
12 months	191	9.7	0.8	5.0	10.0	10.0
24 months	202	9.8	0.7	6.0	10.0	10.0
5 years	204	9.7	0.7	6.0	10.0	10.0
Harris hip score						
PreOP	216	45.6	15.7	7.0	47.0	88.0
6–12 weeks	211	89.0	9.4	48.0	91.0	100.0
6 months	186	96.4	6.1	67.0	99.0	100.0
12 months	191	97.2	5.1	72.0	99.0	100.0
24 months	202	98.2	4.1	72.0	100.0	100.0
5 years	204	97.8	4.7	65.0	100.0	100.0

Radiological analysis in comparison to the 2-year results is summarised in Table 2. Whereas after 2 years bone remodelling in terms of resorption of proximal femoral bone stock was detected in a total of 8 cases (3.9%), at mid-term signs of stress-shielding were spotted in 85 cases (42.3%) (*p* < 0.0001). Again, all of these occurred in Gruen zones 1, 2 and 7 (Table 2). A reduction of radiolucent lines could be observed at mid-term follow-up (3.0% versus 1.0%) (*p* = 0.1025). They occurred exclusively in Gruen zone 1. Signs of femoral cortical hypertrophy remained stable at mid-term follow-up compared to the short-term results with 9 cases (4.5%) (*p* = 1.0). Those were localised exclusively in Gruen zones 3 and 5 (Table 2). Osteolysis was not seen in any patient at any time point.

**Table 2:** Incidence of radiological alterations over time

	2 years		5 years	
	*N*	Percent	*N*	Percent
Resorption femoral bone				
No	195	96.1	116	57.7
Yes	8	3.9	85	42.3
Cortical hypertrophy				
No	194	95.6	192	95.5
Yes	9	4.4	9	4.5
Lucent lines				
No	197	97.0	199	99.0
Yes	6	3.0	2	1.0
Osteolysis femoral bone				
No	203	100.0	201	100.0
Yes	0	0.0	0	0.0

Out of all cases included,15.7% showed measurable subsidence of 2 mm and more after 6 weeks. Only 1.1% of these showed further progression at the next follow-up [20]. Compared to the 2-year results, at mid-term follow-up there was no further progression of subsidence detected in any patients included.

## Discussion

We prospectively evaluated the clinical and radiological outcomes of a cohort of 162 patients who underwent THA with a calcar-guided short stem in 216 hips, demonstrating very good clinical outcomes with high levels of patient satisfaction and 100% stem survival at 5-year follow-up. Whereas radiological alterations such as cortical hypertrophy remained stable at a low level and even a reduction of radiolucent lines could be observed compared to the 2-year results, the incidence of proximal bone remodelling in terms of stress-shielding increased markedly.

Worldwide, an increasing number of young and active patients are treated with THA, who are more demanding regarding postoperative clinical function and physical activity [33]. In Europe, already over 20% of all patients treated with THA are under the age of 60 years [34]. Thus, three main things are required from contemporary THA. Firstly, it should provide good function, to allow for adequate activity, which we expect to achieve using a meticulous, soft-tissue sparing, surgical technique. Secondly, a maximal longevity is desired, which is considered best to be influenced through implant engineering and possibly affected by the patient’s level of activity. Thirdly, the implants must allow for future revision surgery [35]. In this regard, preserving bone stock at the initial surgery seems crucial [1]. Bone loss around cementless femoral components is suspected to precede implant loosening and contribute to issues regarding revision surgery [36].

Recent publications highlight the potential for the avoidance of proximal bone loss with neck-preserving short stems at short-term follow-up [37–39]. The present radiographic analysis at mid-term follow-up suggests that bone remodelling cannot be avoided completely with the design of a calcar-guided short stem. Particularly in Gruen zones 1, 2 and 7 a high incidence of bone resorption was observed at 5-year follow-up. However, the current investigation does not allow for a quantification of the reduction of BMD, thus a comparison to conventional stem designs or different short-stem designs is somewhat difficult. Kress et al., in a study using quantitative computed tomography-assisted osteodensitometry, found a decreased BMD for all Gruen zones only 1 year after implantation of the CFP stem (Link, Hamburg, Germany), with most loss in Gruen zones 1, 2 and 7 [40]. They concluded that the postulated metaphyseal fixation of the CFP stem was not achieved 7 years post-operatively compared to the conventional tapered designed stem, where load is transferred to the distal diaphysis only. The CFP stem, however, achieved a more proximal diaphyseal load transfer. Thus, the goal of the stem-design has been achieved partially [40]. Using the Metha stem (B.Braun/Aesculap, Tuttlingen, Germany) the mean BMD at 12 months post-operatively also decreased in all Gruen zones except for Gruen zones 5 and 6; however, it recovered partially in Gruen zones 1 and 7 without reaching baseline values [4]. Furthermore, in a study using the Nanos stem (Smith & Nephew, London, UK), also providing a calcar-guided design, almost similar results could be observed with decrease of BMD in Gruen zones 1 and 7 and stable conditions in Gruen zones 3, 5 and 6, respectively [41]. The Fitmore stem (Zimmer, Warsaw, IN, USA) shows a different pattern of bone remodelling, with only moderate bone loss at the proximal femur (Gruen zones 1 and 7) but with an increase in density along with cortical hypertrophy in Gruen zones 3 and 5 [42, 43]. This argues for a more distal anchorage due to the stem design. Given distinct differences in the manifestation of bone loss, Yan et al. concluded in a recent review analysis, that short stems should not generally be summarised as one single implant group, as the periprosthetic bone remodelling is highly dependent on the particular stem design [16]. The Metha and Nanos stems show a predominantly metaphyseal anchorage, while the Fitmore and CFP stems offer a more distal load transfer. The Metha stem leads to bone loss in the calcar region, while the Nanos stem revealed bone resorption mainly in the greater trochanteric region, which can be related to the differing stem designs. Nevertheless, compared to conventional stems such as the CLS stem (Zimmer, Warsaw, IN, USA) and the Bicontact stem (B.Braun/Aesculap, Tuttlingen, Germany), the study of Yan et al. also shows that most short stems offer an overall lower rate of bone remodelling [16].

**Fig. 3:**
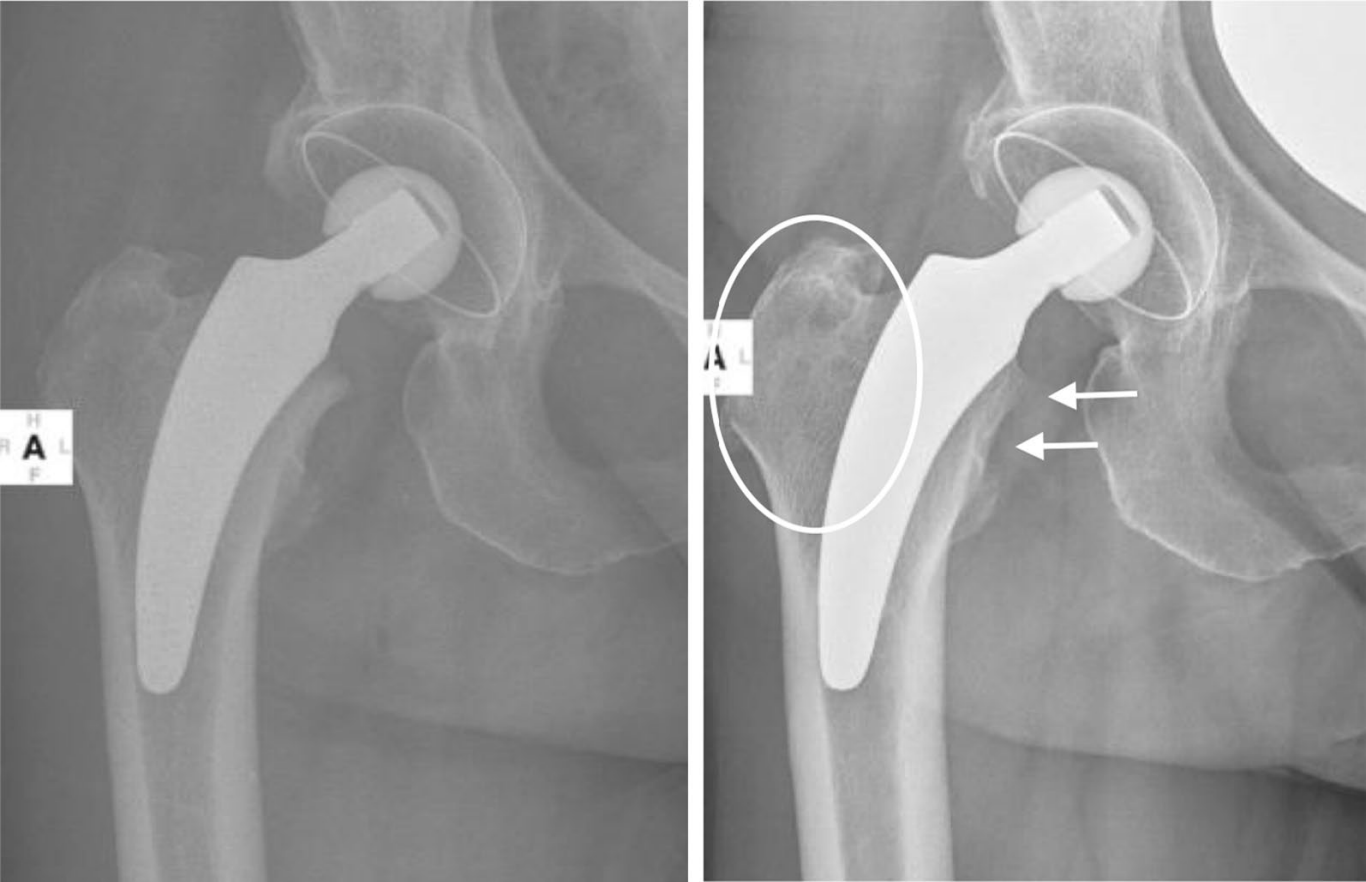
Varus alignment with metaphyseal anchorage (left: postoperative; right: 5-year follow-up). No significant stress-shielding can be found

**Fig. 4:**
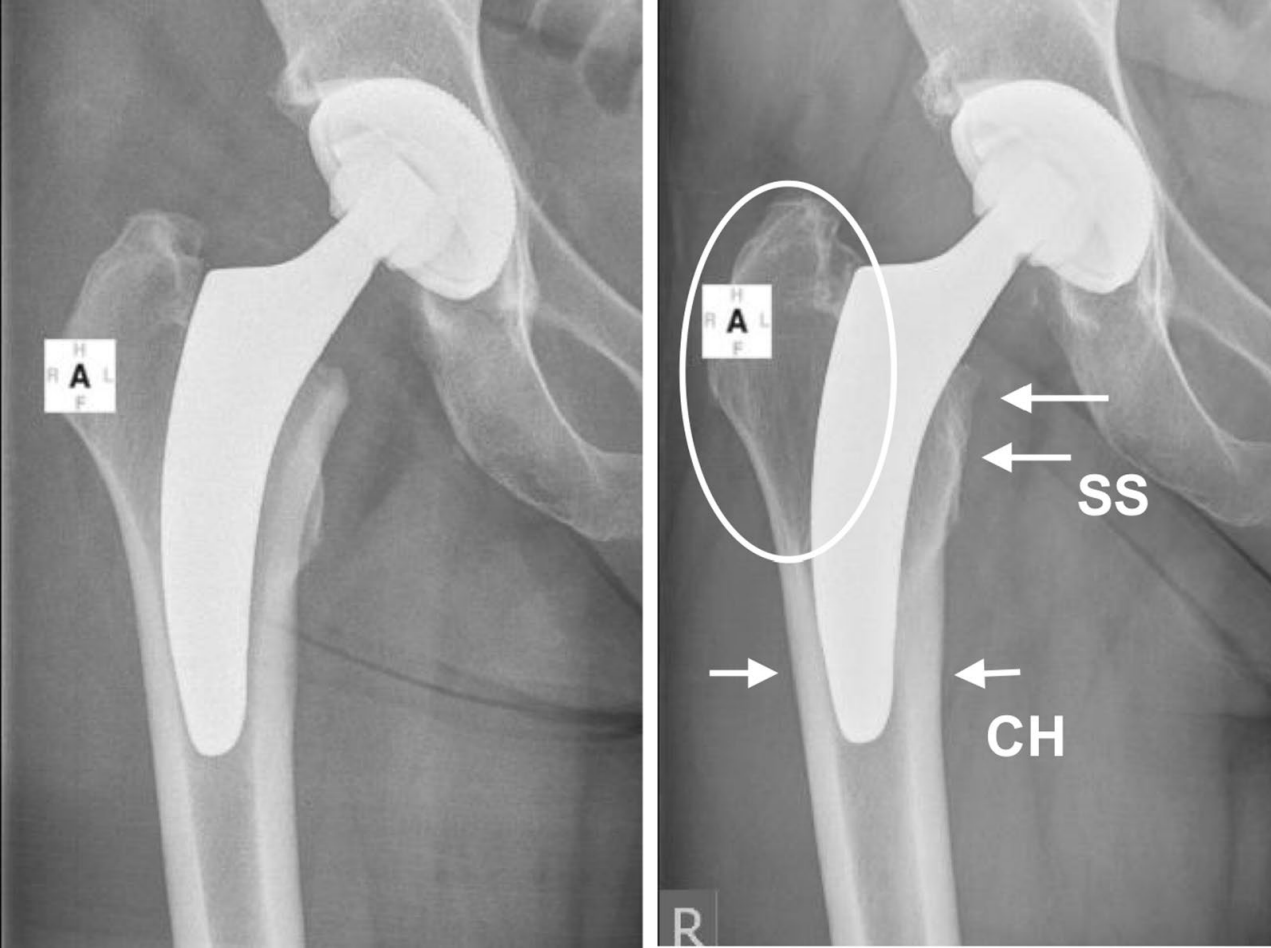
Neutral alignment with additional diaphyseal anchorage (left: postoperative; right: 5-year follow-up) (SS: stress-shielding; CH: cortical hypertrophy). Stress-shielding is obvious in Gruen zones 1, 2 and 7. Cortical hypertrophy can be observed in Gruen zones 3 and 5

The principle of implanting the calcar-guided optimys short stem consists of an individual alignment alongside the medial calcar, providing the ability of reconstructing varus and valgus anatomy in a great variety, using individualised levels of osteotomy [14]. This results in a broad range of CCD angles to be reconstructed but also in different types of anchorage [13, 22]. Whereas most varus hips achieve stabilisation by three-point fixation and fit-and-fill in the metaphyseal area, some neutral and most valgus hips stabilise by supplementing an additional anchorage in the proximal diaphysis. The present investigation suggests continuous stress shielding to be responsible for the proximal cortical and cancellous bone loss observed especially in neutral and valgus alignments with additional diaphyseal anchorage. In varus alignments, fewer signs of proximal bone loss in Gruen zone 7 could be observed (Figs. 3, 4).

Similar to the remodelling around the Nanos stem, in the current investigation the major bone resorption was observed in the greater trochanteric region (Gruen zone 1) and is most likely related to the calcar-guided load-transfer given by the implant alignment in varus hips (Fig. 3). In contrast, very little remodelling in terms of stress-increase occurred in the distal regions (Gruen zones 3, 4 and 5), which would correlate with the clinical absence of thigh pain and low incidence of cortical hypertrophy [20] (Fig. 3). In neutral or valgus hips, the reduction in BMD additionally occurred mainly in Gruen zone 7, in a few cases accompanied by cortical hypertrophy in Gruen zones 3 and 5 (Fig. 4).

Thus, it is likely that not only the type of implant design has to be considered crucial regarding different patterns of bone remodelling, but also the stem alignment. This corresponds to previously published literature. Parchi et al., in a DXA study investigating the Metha stem, also came to the conclusion that bone remodelling is related to the surgical technique and the final implant position [3]. Brinkmann et al., analyzing the Metha and the Nanos stem as a function of varus and valgus stem positioning regarding bone remodelling, found moderate stress-shielding of the proximal femur with pronounced but not exclusive metaphyseal loading. In their investigation, varus positioning also led to increased medial loading with reduced stress-shielding. Valgus alignment induced loss of BMD in the medial proximal femur and increased distal load transfer [44]. However, the amount of change was small.

Theoretically, the resorption of periprosthetic cancellous bone may contribute to mid-term migration, the rate of which has been linked to subsequent failure and to the process of aseptic loosening [45]. However, although some loss of proximal bone density could be observed in almost half of the investigated cases, no further axial migration compared to the 2-year results, in varus hips as well as in valgus alignments, was observed. This corresponds to the recently published migration analysis after 24 months using Einzel–Bild–Roentgen-Analysis (EBRA-FCA) [24]. Additionally, given a 100% survival rate of the investigated stem in the present cohort at mid-term, the findings seem to be of little clinical relevance at present.

Limitations have to be acknowledged. The radiological method used to evaluate femoral bone remodelling is rather inaccurate compared to the usage of the DXA method and does not allow quantification of bone loss. However, given the size of the cohort, DXA scans would have resulted in intense effort and costs. Future investigations will have to verify the results. Also, the method of measuring axial migration lacks accuracy; however, previously published EBRA-FCA analyses confirm the findings. Thirdly, a control group is lacking given the present study design. Future studies should focus on comparing calcar-guided short-stem THA with conventional THA.

The present study, to our knowledge, is the first one to provide mid-term results for the specific design of the optimys stem.

In conclusion, very good clinical outcomes resulted in high levels of patient satisfaction at mid-term follow-up. To date no stem revisions have had to be performed, resulting in a survival-rate of 100%. No further axial migration could be observed. Future investigations should evaluate whether these results can be maintained in a long-term follow-up.

The intended preservation of bone stock can only be accomplished partially using this particular stem design. Depending on the stem alignment, an additional diaphyseal anchorage, besides the metaphyseal fixation, may result, potentially leading to some amount of stress-shielding and remodelling of the medial proximal femoral bone comparable to diaphyseal anchoring conventional stems. Future DXA studies are needed to confirm the present results. The clinical relevance of those measurements remains to be proven.

## Data Availability

The datasets generated and/or analysed during the current study are not publicly available due to the high volume of data but are available from the corresponding author on reasonable request.

## Abbreviations

THA: total hip arthroplasty
EBRA-FCA: “Einzel Bild Roentgen Analysis”-Femoral Component Analysis
RSA: radiostereometric analysis
CCD: caput-collum-diaphyseal-angle
HHS: Harris hip score
VAS: visual analogue scale
BMD: bone mineral density
DVT: deep vein thrombosis
DXA: dual-energy X-ray absorptiometry
SD: standard deviation
FU: follow-up
